# Amalgam for Filling Carious Teeth

**Published:** 1870-08

**Authors:** W. Geo. Beers

**Affiliations:** Dentist, Montreal; Clinical Lecturer to Royal College of Dental Surgeons of Ontario; Honorary member of Ontario Dental Society; Secretary of Dental Association of Quebec, Corresponding Member of Odontographic Society of Pennsylvania; Editor of Canada Journal of Dental Science, &c.


### ARTICLE IX.

### *Amalgam for filling Carious Teeth.*

By W. Geo. Beers, Dentist, Montreal;

Clinical Lecturer to Royal College of Dental Surgeons of Ontario; Honorary member of Ontario Dental Society; Secretary of Dental Association of Quebec, Corresponding Member of Odontographic Society of Pennsylvania; Editor of Canada Journal of Dental Science, &c.

In the January number of the *Canada Medical Journal* appears an article by Mr. Bowker, dentist, on the use of amalgam for filling carious teeth, which calls for some reply. The author "assumes to be the expositor of the abuses of dentistry," and has the modesty to intimate that he stands almost alone as a Canadian dentist who does not adhere to a practice which he calls "unskillful, ignorant and dishonest," and the assurance to identify with this practice our Canadian Dental College and Societies, and the private practice of almost all dentists in the Dominion. I do not remember an other instance in your Journal where a correspondent, discussing a scientific question, found it necessary to impute inability, ignorance or dishonesty to all who were not of his opinion. I find no fault with Mr. Bowker for the facility with which he attempts to dispose of a question involving abstruse chemical, physiological and pathological causes and effects, and necessitating extensive research and experiment into the particular subject; but I cannot see the necessity for unjust accusations to give force to scientific argument. Especially when one essays to be a philanthropist, he should, at least condescend to be correct.

Mr. Bowker asserts that the "American Society of Dental Surgeons" *unanimously* carried a resolution (in 1845) condemnatory of amalgam. That is wrong. Sixty-one of the one hundred and thirty-three members voted against it, and *the resolution was finally rescinded by the very majority who framed it.*

Mr. Bowker says; "The institution *with the imposing title* of 'Royal College of Dental Surgeons' encourages the use of amalgam' and that the same may be said of the 'Dental Association of Quebec;' and "that the Dental Societies of Canada, who put themselves forward as the guardians and representatives of the profession in the Dominion, not only advocate but vindicate its use." It is not surprising that your correspondent is ignorant of what has or has not really transpired in our dental Societies, &c., as he is not a member of any; but as an illustration of the trouble he has taken to make himself acquainted with facts, I am qualified to assure you that neither college nor societies have once, directly, or indirectly, discussed the subject, and that all clinics in "filling teeth" have invariably been done with gold. The sneering allusion, *en passant*, to the College may pass for its full value, but I venture to say that the institution will survive any such disparagement. On its faculty it has some leading medical men of Ontario, such as Drs. Canniff, Sangster, Rolph, Bethune, Geike, Berryman, &c., as well as leading dentists of the upper Province, and, moreover, it, with the societies and the progressive movements generally of the profession, have the hearty sympathy of all prominent medical men in Ontario.

Another assertion is that "the highest dental and medical authorities European and American, have condemned the use of amalgam, in any form whatever, for filling teeth, as malpractice.', Now, we must expect differences of opinion upon this whole subject of amalgam ; but no reasonable man will say that opinions dictated when amalgams were composed of impure metals, and imperfectly understood, can continue to hold good against the superior improved article. The possibility of even a good amalgam being abused by the use of impure mercury, or "sixty-four parts of mercury to thirty six of silver," is no more reasonable argument against the use of this material, than an argument that no preparation of arsenic, morphia,, &c., should be used because they are infallible poisons. To-day there are some dental writers whose opinions of amalgam may be quoted as argument against its use; but Harris, Piggot and others wrote when amalgam was abused, and in the former work we find methods of "treatment" recommended for various dental diseases, something analogous to amputating a finger for a wart. And it must be remembered that the names "Royal Mineral Succedaneum," &c., transcribed by Mr. Bowker, were only given to the old compounds many of which contained copper, lead, bismuth and other alloys, and that these old compounds were decidedly "condemned" by all intelligent dentists, and are to-day. But when the material was improved, and brought to the pureness of the amalgams of the present time, many of the very leaders who opposed "Royal Mineral Succedaneum" approved of and used the improved compound.

We might give up filling teeth altogether if we were to abstain from every material that may be "abused." A dentist whose gold fillings are soft as cheese a month after insertion gives us clear demonstration either of his "unskilfulness, ignorance, or want of honesty;" and certainly any one who would put an amalgam into a tooth which had 64 parts of mercury to 36 of silver would be deserving of some much stronger epthet than "dishonest." The use of filthy amalgam is paralleled by the use of impure gold; slovenly manipulation with either material has the same results. Teeth are filled with gold and amalgam that should never be filled at all, but alveolar abscess resulting after the latter is traced by some to the material, when a worse case after the use of gold is diagnosed to causes distinct from it. There are certain conditions of dental caries where we can guarantee to produce periosteal disease by inserting a gold or any other filling; and amalgam, unfortunately for its reputation, is too often used as a *dernier resort* in teeth which should never be filled at all.

Before giving your readers brief extracts from the latest opinions of "high authorities" in Europe and America, I may venture to say, with the highest respect for the knowledge of those who are not dentists, and who have given opinions prejudiical to amalgam, that the opinions upon this subject of men of acknowledged ability and experience, who, besides being physicians and practical chemists, are eminent dentists, are worth something more upon the average, than the opinions of men who have not the same special life long experience with the pathology of the teeth. For over twenty-five years this subject has been before the dental profession; every day, we may say every hour fresh opportunities appear for investigation: and the opinions of such investigators as I will quote surely cannot be ignored.

No one in Europe has made more thorough and dispassionate investigations into this subject than the author of "Tomes' Dental Surgery," a name familiar to more than dentists. In the early editions of his "Surgery" he says he never saw a case of salivation, and did not believe there could be such a result from the use of amalgam. A few years ago he made more extensive researches, and reiterated his former conclusions more forcibly than ever. I might quote, *in extenso*, from English, German and French authors of the highest repute, who approve of and use amalgam. I

am not disposed to believe that the State Dentists of Europe, and such men as Tomes, Naysmith, Saunders, &c., are prompted to use amalgam by any of the considerations Mr. Bowker asumes, viz.: "cheapness, want of skill, ignorance, or the want of honesty."

Nothing, however, more conclusively reveals the truth than the facts that, with some few exceptions, the American dentists, who so violently opposed amalgam in 1845–47, now use it. Some who even continue to condemn it, admit that they do so, because there is a danger of it being abused, while some few still maintain the same objections to the improved amalgam that they held towards "Royal Mineral Succedaneum." To-day you may go into dental offices in New York, and witness the operator filling teeth from a pot of amalgam, which is mixed and kept ready for use at all hours! (Sundays not excepted.) I presume the proportions are 64 parts of mercury to 36 of silver. Now because there is this abuse of the material, that is neither logical nor scientific argument against its proper use.

Dr. Pierce late Professor of Dental Physiology and Operative Dentistry in Pennsylvania Dental College, says; "Many attribute results to amalgam that are due to its improper use. That there are teeth that can be saved for a much longer period, and with greater advantage to the possessor with amalgam, than with foil, especially as some dentists insert foil, no unprejudiced practitioner can deny."

Dr. Buckingham. Dean and Professor of Chemistry in Pennsylvania College, says "he uses amalgam in his own practice, and thinks a dentist to do justice to his patients, should use it in certain cases. Had never seen a case of salivation from it, and had strong doubts of it ever producing ptyalism."

Dr. McQuillen, Dean and Professor of Physiology in Philadelphia Dental College, and editor of the well-known "Dental Cosmos," says "He had had strong objections to amalgam, and had opposed its use; but candor compelled him to say that his views had changed, and he does not now regard it as the unquestionable cause of all the difficulties ascribed to it; for in an experience of fourteen years he could not recall a single instance of ptyalism &c., of which others asserted they had seen so many. He looked upon those who had asserted to have seen them, with considerable doubt as to the value of their judgement and opinions as reliable diagnosticans."

Dr. Fitch a leading physician and dentist of New York "pleaded guilty to the charge of early prejudice against amalgam. He was now, however, disposed to give it its appropriate place, even at the risk of encouraging empiricism and cheap dentistry. He had yet to see the first case of ptyalism from its use."

Dr. Flagg, Professor of Dental Pathology and Thera. in Philadelphia Dental College, and *the first man who wrote against amalgam in* 1845, says, "he is now entirely opposed to the statements made regarding the constitutional effects from amalgam fillings. He had again and again treated teeth suffering from periostitis, &c., attributed to amalgam, and had refilled them with amalgam, for the sole purpose of proving to his patients that the material used had nothing to do with the trouble existing."

Dr. Allen, formerly editor of the "Dental Record" has written folios to expose the fallacious arguments of the opponents of amalgam. He says: "I have seen over 1000 persons, each having from 1 to 10 amalgam fillings in their teeth, and I only saw one case of ptyalism, which I attributed to dead and ulcerated roots. As soon as the roots were removed the trouble gradually disappeared. I have been brought to see cases of supposed ptyalism from amalgam fillings and have found them to be nothing of the kind, but owing the origin of the disease to salivary calculus, ulcerated roots, sponginess of the gums, &c."

Dr. Garretson, author of a late work of 700 pages on "Diseases and Surgery of the mouth, &c," describes the mode of using amalgam for filling teeth, and totally ignores the charges made against it.

I might continue thus to quote from men whose ability to investigate, opportunities to examine, and integrity to report conscientiously, cannot for a moment be doubted. The opinions cited above are founded upon thorough experiment and extensive experience; their authors have their attention confined to the mouth, and the opinions of such men cannot be ignored. The February number of the "American Journal of Dental Science" contains a review of this very article of Mr. Bowkers, in which it says: We think Mr. Bowker has taken an extreme view of the case. *We think this compound may be used* in teeth which are mere shells so far gone that no other metal can be safely introduced. *When properly prepared and properly introduced*, instead of amalgam containing 64 parts of mercury to 36 of silver as Mr. Bowker asserts, the proportions of mercury need not and should not be half so great." It proceeds to give "*the best* method for using this material." Is that a condemnation of amalgam?

I do not find one single argument in Mr. Bowker's paper that was not known and proclaimed a quarter of a century ago; the names he ascribes to it are a quarter of a century old, and posthumous; the proportions he gives are only those of the quacks, Crawcours, who introduced a filthy amalgam into American dentistry over 30 years ago, and who have been dead nearly twenty! Precisely the same arguments Mr. Bowker put forth may be found in the early volumes of American Dental Journals, and, strange to say, some parts of his paper are word for word, saving one or two alterations such as "teeth" for "mouth," &c., with an essay on amalgam by Dr. Geo Watt. (See Watt's Chemical Essays, page 149.)

The charge against amalgam of producing ptyalism is, I hold, mere assumption. The astute pathological knowledge of those who write against the material, is seldom capable of giving an opinion so precise upon any other disease in any other part; and their utter disregard of any other cause of salivation, when there is amalgam in the teeth, is very expeditious, if not very sound. We know that idiopathic or spontaneous salivation may, and does arise where no mercury has been used; and that the same symptoms of swelling of the salivary glands. fetor, &c., may be present; and that certain preparations of gold, antimony, &c., will produce profuse salivation. Watson, in his Practice of Physic names castor-oil, digitalis, iodide of potassium, opium, pregnancy, &c., as possible causes of ptyalism; but the opponents of amalgam, if they find it in the teeth, at once pounce down upon that unhappy material as the sole and only cause. I should hardly venture to call that scientific.

The following concise opinion by a "high authority"—who is a physician, dentist and practical chemist—is preferable to words of my own. "Certain *preparations* of mercury, it is well known, produce, under certain circumstances, very powerful effects on the human frame; but in order to do so, it requires to undergo a change in its composition and character, which in the form in which we find it in amalgam, it never does nor can undergo. It needs a degree of oxydation or a minuteness of division which renders it useless for filling. The two characters are inconsistent with each other. If the quicksilver in the mixture undergoes such a change as to allow of its mercuralizing the system, it will not answer as an ingredient in the filling, and *vice versa*. In the combinations it forms in this filling, and as long as it remains so combined, it cannot effect the system. Before the system can become subject to its mercuralizing influence it must first become uncombined from the silver, which is impossible because of its stronger affinity for that metal than for oxygen; and secondly, must recombine with such a quantity of oxygen, as it cannot be made to unite with it in the mouth. Add to this, that the exposed surface of the filling itself must also have lost its cohesion; to have become quite soft; and to have very perceptibly diminished in quantity. But in the face of these impossible, yet absolutely necessary changes, we find amalgam fillings still solid and sound after being in the mouth ten and twenty years, and not a single symptom of mercurial action, local or general, upon the system!

With equal propriety it might be urged against gold, that because, when highly oxydized, it becomes a powerful medicinal agent, therefore it should not be used as a filling for teeth. The error of those opposed to amalgam proceeds from a want of discrimination between diseases totally different in their cause, character and consequences."

It is very strange and suggestive that out of the thousands of times and for many years in which amalgam has been used, and in many instances abused, that so few cases of local or constitutional disease arising from its use are heard of; and it must be remembered that, if such evil results ensued from amalgam, it is not likely they would entirely escape the observation of such intelligent and responsible men as those I have quoted. It is equally strange and suggestive that a case of such supposed disease was never met with by any but those who opposed amalgam; and that not a single new argument against its use has been adduced for a quarter of a century, while, on the other hand, the quality of the material has been vastly improved, and the importance of careful manipulation thoroughly understood.

In concluding this hasty review of Mr. Bowker's article, I trust I will not be misuuderstood, and here, I think, I may venture to speak for a large majority of Canadian dentists who use amalgam. The proceedings of our Dental Societies will convince you that "gold" is acknowledged to be, by far, the best material for filling teeth for the large majority of cases; but there are circumstances and occasions when gold or tin foil cannot be used to the best advantage. Those who oppose amalgam admit this, and what do they use instead? Preparations of gutta-percha, "Osteoplastic" and other such soft fillings. The former will soften in hot water, and neither can preserve the teeth intact more than a few months. With the greatest care to use only the best amalgam, the purest mercury, to thoroughly remove every particle of decay from the tooth, and prepare it as carefully as if for gold, and see that the proportion of mercury is not 64 parts to 36 of silver, we hold that amalgam arrests decay, and cannot cause either local or constitutional trouble of any kind. On the other hand, we freely acknowledge the possibility of injurious results from Mr. Bowker's proportion of 64 of mercury to 36 of silver, especially if the compound is old "Royal Mineral Succedaneum." Even the best amalgam has its place; it is kept there. Those who use it in the front teeth, and for small cavities anywhere, are quacks; but those whose *gold* fillings as soft as cheese a month after insertion are not much better.

I regret that Mr. Bowker's unusual style of discussing a scientific question has compelled me to skim over practical points; but his unjust accusations have goue to the public, and being unjust it is but right that they should be refuted. Such a modest syllogism as the following—which is simply reduced from his own words—cannot be permitted to pass without a censure, or at any rate, a joke.

Whoever uses amalgam is "unskilful, ignorant or dishonest."

The Canadian Dental College and Societies, and "almost all dentists in the Dominion use amalgam," and I do not.

Therefore, "almost all Dentists in the Dominion" are "unskilful, ignorant or dishonest," and I am not.—*Canadian Medical Journal.*

